# Rapid Monitoring of Organochlorine Pesticide Residues in Various Fruit Juices and Water Samples Using Fabric Phase Sorptive Extraction and Gas Chromatography-Mass Spectrometry

**DOI:** 10.3390/molecules24061013

**Published:** 2019-03-13

**Authors:** Ramandeep Kaur, Ripneel Kaur, Susheela Rani, Ashok Kumar Malik, Abuzar Kabir, Kenneth G. Furton, Victoria F. Samanidou

**Affiliations:** 1 Department of Chemistry, Punjabi University, Patiala 147002, India; ramandeep.chem@gmail.com (R.K.); ripneel83chahal@gmail.com (R.K.); susheela.chemistry@gmail.com (S.R.); 2 Department of Chemistry and Biochemistry, International Forensic Research Institute, Florida International University, 11200 SW 8th St, Miami, FL 33199, USA; furtonk@fiu.edu; 3 Laboratory of Analytical Chemistry, Department of Chemistry, Aristotle University of Thessaloniki, 54124 Thessaloniki, Greece; samanidu@chem.auth.gr

**Keywords:** fabric phase sorptive extraction, gas chromatography-mass spectrometry, organochlorine pesticides, sample preparation

## Abstract

Fabric phase sorptive extraction, an innovative integration of solid phase extraction and solid phase microextraction principles, has been combined with gas chromatography-mass spectrometry for the rapid extraction and determination of nineteen organochlorine pesticides in various fruit juices and water samples. FPSE consolidates the advanced features of sol-gel derived extraction sorbents with the rich surface chemistry of cellulose fabric substrate, which could extract the target analytes directly from the complex sample matrices, substantially simplifying the sample preparation operation. Important FPSE parameters, including sorbent chemistry, extraction time, stirring speed, type and volume of back-extraction solvent, and back-extraction time have been optimized. Calibration curves were obtained in a concentration range of 0.1–500 ng/mL. Under optimum conditions, limits of detection were obtained in a range of 0.007–0.032 ng/mL with satisfactory precision (RSD < 6%). The relative recoveries obtained by spiking organochlorine pesticides in water and selected juice samples were in the range of 91.56–99.83%. The sorbent sol-gel poly(ethylene glycol)-poly(propylene glycol)-poly(ethylene glycol) was applied for the extraction and preconcentration of organochlorine pesticides in aqueous and fruit juice samples prior to analysis with gas chromatography-mass spectrometry. The results demonstrated that the present method is simple, rapid, and precise for the determination of organochlorine pesticides in aqueous samples.

## 1. Introduction

Organochlorine pesticides (OCPs), a sub-class of persistent organic pollutants (POPs), have been mass-produced since the 1940s and widely applied in agriculture worldwide as important insecticides because of their cheaper price. They gained popularity due to their effectiveness in controlling mosquitoes, hence controlling malaria and typhoid fever [1,2]. OCPs are among nine of the initial “dirty dozen” persistent organic pollutants (POPs) identified by the Stockholm Convention on persistent organic pollutants (POPs) in 2001 [3]. As OCPs have low water solubility and high lipid solubility, they easily accumulate in the environment and living organisms. Additionally, these persistent environmental pollutants are very hazardous, and due to their volatility are susceptible to long-range atmospheric transport [4,5]. They are capable of biomagnifications through the food chain. Surface runoff from non-point sources, discharge of industrial wastewater, disposal of empty containers and equipment, discharge from surface application of pesticide [6] and careless washing lead in the addition of OCPs to the aquatic environment [7]. A study revealed that OCPs are responsible for toxic effects to aquatic organisms. Furthermore, they can accumulate in the ecosystem and potentially pose threat to biodiversity [8]. Despite the ban on their industrial production since the 1970s, OCPs are still found at trace levels in the environment due to their resilient physiochemical properties. OCPs have been found to cause several carcinogenic and non-carcinogenic disorders in humans [9,10]. Increasing concern regarding health safety issues has emphasized the need for detection of pesticides at trace levels in the drinking water and food as these have leached the soil and entered the food chain [11]. Therefore, detection of OCP residues is of utmost importance in estimating potential health risks, performing ecotoxicological risk assessments, and enforcing regulations [12,13]. The development of simple, fast, reliable and environmentally friendly methods that enable the determination of OCPS at trace levels in aqueous samples is of great concern nowadays [14,15].

For the trace analysis of target analytes in various matrices, the sample preparation is of paramount importance to analytical efficiency and analyte recovery. Various extraction techniques based on solid sorbents have been extensively used for the determination of OCPs from aqueous samples including solid-phase extraction (SPE) [5,16], solid-phase microextraction (SPME) [17], single drop microextraction (SDME) [18], magnetic solid-phase extraction (MSPE) [19], micro solid-phase extraction (µ-SPE) [20,21] and stir-bar sorptive extraction (SBSE) [22,23]. The extraction of analytes relies on the type of sorbent material being used. Moreover, selectivity of the extraction method depends upon the nature of the sorbent, which determines their affinity towards the target compounds. In this regard, sol-gel technology has achieved considerable success in analytical sciences, with potential applications in the adsorption and separation of various analytes which result from their distinct features, such as unique selectivity, enhanced extraction sensitivity and higher thermal, mechanical and solvent stability [24]. FPSE, introduced in 2014 by Kabir and Furton, represents an important development for the extraction of several organic pollutants at trace and ultra trace levels. It combines the sampling, isolation and enrichment of the analytes in a single step, making it a quick sample preparation process [25,26]. FPSE consists of a small cellulose or polyester fabric coated with a thin layer of a suitable sorbent phase by sol-gel coating technology. There is a large number of coatings available for FPSE, and the extraction performance is dependent on the choice of an appropriate sorbent [27,28]. FPSE is an equilibrium technique based on partitioning of the analytes between the matrix and an extraction phase. The FPSE media is inserted into the sample solution, which is agitated for a certain time, and the adsorbent with the adsorbed analyte is then separated from the solution [29]. The analytes are consequently eluted and analyzed. FPSE possesses some exceptional and unprecedented properties such as ultra-high specific surface area, increased surface activities, flexible functionalization and tunable composition, which make them suitable for versatile and efficient sample preparation [30,31,32]. Additionally, FPSE media are very stable in a wider range of harsh organic solvent due to stronger chemical bonding between sol-gel sorbent and fabric media [33]. One of the most crucial factors for successful application of FPSE is direct extraction of analyte from real sample matrices without any specific requirement, such as filtration, centrifugation, solvent evaporation and sample reconstitution [34,35,36].

The present study involved an exploratory study on the feasibility of using FPSE media for the extraction and determination of persistent organic pollutant in various real sample matrices. This paper demonstrates a simple, rapid and efficient FPSE method coupled with GC-MS that we have developed to analyze the amount of organochlorine pesticides in water and juice samples obtained from market. The optimization of the analytical process, including the influence of several experimental parameters, selectivity, and interaction mechanisms, is fully discussed.

## 2. Results and Discussion

### 2.1. Optimization of Fabric Phase Extraction

To evaluate the capability of FPSE media, the extraction of a mixture of organochlorine containing 19 OCPs as model compounds from water and juice samples were investigated. Several FPSE parameters, including the extraction time, stirring speed, desorption solvent and its volume, desorption time, and ionic strength, were investigated to achieve the best extraction efficiency of the chosen FPSE media.

#### 2.1.1. Selection of Fabric Phase Sorptive Extraction Sorbent Chemistry

**Figure 1:**
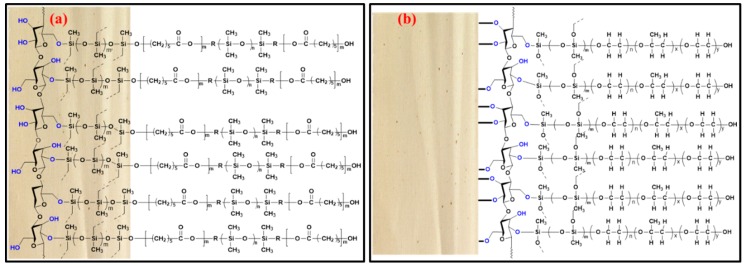
Schematic representation of (**a**) sol-gel PCAP-PDMS-PCAP; (**b**) sol-gel PEG-PPG-PEG coated FPSE media.

One of the most important tasks in fabric phase sorptive extraction method development is to select the appropriate sorbent chemistry that would offer the highest selectivity and extraction efficiency towards the target analytes. Failure to select the appropriate sorbent chemistry may substantially limit the overall sensitivity of the analytical method. Unlike other microextraction techniques, which have a limited number of sorbents to choose from, fabric phase sorptive extraction offers a large number of sol-gel-based high-efficiency sorbents that can potentially be used for a given application. It would be an utterly tedious job if one had to test all the available sorbents, as in the case of most sample preparation techniques, to find the most suitable one. To simplify the sorbent selection process, fabric phase sorptive extraction has developed an absolute recovery prediction calculator using the logKow values of the analytes for each of the FPSE sorbent media. Using the logKow value of the analyte, one can predict about the tentative absolute recovery of the particular analyte. For multi-residual analysis, once the recovery value for each of the analyte is calculated, the FPSE sorbent, which provides the highest recovery values for most of the analytes, can be selected or two/three FPSE sorbent media, can be short-listed for method development and the best one can be selected from the experimental data. Taking the medium and low polarity of the organochlorine pesticides into consideration, two medium polar FPSE coatings, sol-gel poly(ethylene glycol)-poly(propylene glycol)-poly(ethylene glycol) (sol-gel PEG-PPG-PEG) and sol-gel poly(caprolactone)-poly(dimethyl siloxane)-poly(caprolactone) (sol-gel PCAP-PDMS-PCAP) were selected as the potential sorbent candidates. The schematic representation of sol-gel PEG-PPG-PEG and sol-gel PCAP-PDMS-PCAP coated FPSE media are presented in Figure 1.

The absolute recovery equations for both the sorbents are given below:

Absolute Recovery, % for sol-gel PEG-PPG-PEG: −3.68 + 23.07 logKow−1.72 (log Kow−2.74)^2^

Absolute Recovery, % for sol-gel PCAP-PDMS-PCAP: −3.36 + 22.34 logKow−2.12 (log Kow−2.74)^2^

Since the model is valid between logKow 0.3–5.07, the predicted absolute recovery values have been calculated for only 7 OCPs whose logKow values are within this range. The predicted absolute recovery values are presented in Table 1.

**Figure 2:**
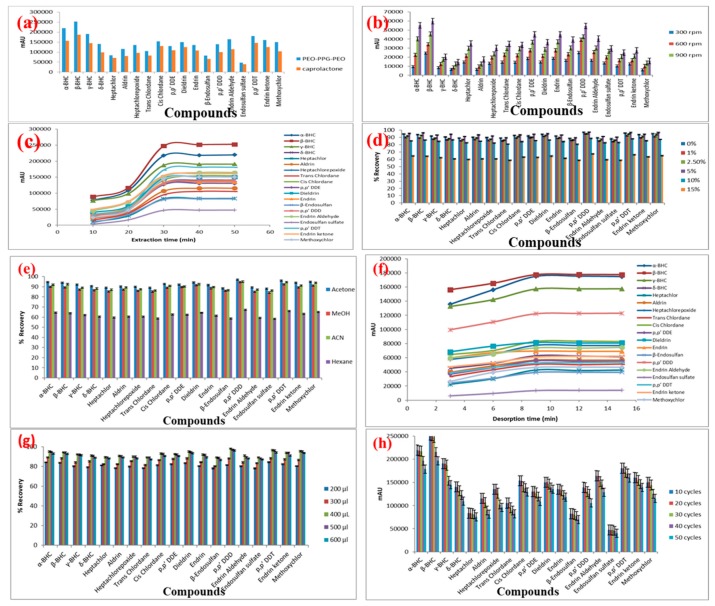
Effect of (**a**) sorbent type; (**b**) agitation speed; (**c**) extraction time; (**d**) salt addition; (**e**) desorption solvent; (**f**) desorption time; (**g**) desorption solvent volume; (**h**) repeated use of FPSE media on the microextraction efficiency of the analytes (sample volume: 10 mL; concentration of the analytes 5 ng/mL).

Both the FPSE media were used for the extraction of the target OCPs from 10 mL spiked aqueous solution at 5 ng/mL, extraction time, 20 min; stirring speed at 900 rpm, back-extraction in acetone for 15 min. The results are presented in Figure 2a. Although the absolute recovery model predicted that sol-gel PCAP-PDMS-PCAP would perform better than sol-gel PEG-PPG-PEG, in reality, the extraction performance for all OCPs were superior in sol-gel PEG-PPG-PEG FPSE media. The root cause for this discrepancy needs further investigation and perhaps refinement of the absolute recovery calculator. Nonetheless, the models can definitely help in shortlisting suitable FPSE sorbents for selecting the best from real experimentations. Based on the experimental results, sol-gel PEG-PPG-PEG coated FPSE media was selected as the optimum sorbent for OCPs and was used in all the subsequent method development and validation experiments.

#### 2.1.2. Effect of Stirring Speed

Sample agitation is a critical parameter in the extraction process according to mass transfer theory. Sample agitation increases the movement of analytes to fabric surface with reduction in thickness of boundary layer and shortened thermodynamic equilibrium time. Therefore, the effect of stirring speed on the extraction efficiency of the analytes was investigated within the interval of 300–1200 rpm. The extraction efficiency of the analytes increased with increase in the stirring speed as demonstrated by the results (Figure 2b). The highest extraction efficiency was achieved at a stirring speed of 1200 rpm. Therefore, 1200 rpm stirring speed was selected for the subsequent experiments.

#### 2.1.3. Effect of Extraction Time

The extraction time is a critical parameter in the FPSE procedure as it greatly influences the partition of the target analytes between the sample solution and the sorbent. FPSE is an equilibrium-based technique, and the mass transfer of analytes from the sample solution to the sorbent is directly influenced by the extraction time. To achieve the highest extraction recovery, the effect of time on the extraction efficiency was examined in the range of 5–50 min. The adsorbed amounts of OCPs generally increased with extraction time up to 30 min with no obvious change occurring thereafter as depicted in Figure 2c. Thus, the extraction reached the equilibrium at 30 min. Consequently, an extraction time of 30 min was selected for further analysis.

#### 2.1.4. Effect of Matrix pH

OCPs are persistent organic pollutants that are present in the neutral state within the entire pH range in an aqueous solution. Hence, the pH of the sample solution was not expected to have a significant impact on the extraction efficiency. Nonetheless, the effect of pH on recovery was conducted by varying pH values ranged from 2 to 10 adjusting with HCl or NaOH (*n* = 3). Indeed, no significant difference in terms of extraction efficiency was observed for OCPs. Hence, subsequent experiments were conducted with measured pH values in the range of 6.0–7.0 without any pH modification in the sample solution.

#### 2.1.5. Effect of Salt Addition

The addition of salt can enhance the ionic strength of the sample solution and diminish the solubility of analytes in the sample solution by salting out effect. Thus, the partitioning of the analytes from the sample solution to the adsorbent is revamped. In this study, the effect of ion strength on the extraction efficiency of the analytes was investigated by adding different concentrations 0, 1, 2.5, 5, 10 and 15%, (*w*/*v*) of NaCl into the standard working solution with three replicates at each point. The results revealed that the addition of NaCl up to 5.0% (*w*/*v*) increases the extraction efficiency due to the salting out effect and then decreases. Further increase in NaCl concentration can increase density and viscosity of the aqueous solution that diminishes the salting-out effect and results in low mass transfer and extraction efficiency (Figure 2d). Thus, 5.0% (*w*/*v*) was chosen as the suitable amount of NaCl for the subsequent experiment.

#### 2.1.6. Desorption Conditions

The selection of an appropriate desorption solvent is necessary for retrieving analytes entrapped in the FPSE membrane to achieve higher extraction recovery. The process of desorption was carried out using different organic solvents, including acetonitrile, methanol, *n*-hexane and acetone. Acetone was proven to yield the highest recovery of analytes by desorbing the membrane immersed in 500 µL of solvent as shown in Figure 2e. Therefore, acetone was selected as the eluent for subsequent experiment. Thereafter, the minimum time required for the complete desorption of the analytes from the sorbent was also investigated in the range of 3–15 min. The extraction efficiency was the highest with adsorption time of 9 min. There was no improvement in the amount of OCPs present in the enriched solvent that was observed when desorption time was prolonged further to 15 or 20 min. Consequently, 9 min was selected as the optimal desorption time for subsequent experiments (Figure 2f).

To evaluate the effect of the solvent volume for complete desorption of the analyte on the extraction recovery, the volume of six different solvent volumes of acetone containing 10 ng/mL OCPs were investigated using the proposed approach. As shown in Figure 2g, the analytical signals for all the OCPs increased and reached their optimum levels when the solvent volume increased from 100 to 400 µL. The extraction sensitivity reduces when the solvent volume was further increased to 500 and 600 µL. Based on the results obtained, a desorption time of 9 min was selected for the subsequent analysis with desorption solvent volume at 400 µL.

#### 2.1.7. Stability and Reusability of Sol-Gel FPSE Media

The stability and reusability of sorbent are the crucial features for better performance of the sorbent. For this purpose, the extraction efficiency, reusability and stability of the sorbents were assessed through thirty consecutive cycles of adsorption/desorption for OCPs extraction.

The stability of the sol-gel PEG-PPG-PEG coated FPSE media was evaluated based on reproducibility of extraction efficiency with different batches of sorbent. The reproducibility of the extraction was examined using five different batches of sol-gel PEG-PPG-PEG sorbent-coated FPSE media with water samples spiked with 5 ng/mL of each OCP. RSD values lower than 6% were obtained, indicating a good reproducibility in the sol-gel sorbent coating process. The regeneration of the FPSE media (for multiple use) was examined with a random batch. After the completion of each extraction-desorption cycle, the used FPSE media was regenerated by rinsing it with 0.5 mL of water and acetone to remove any residual or other substances. The regenerated sorbent was then inserted into the glass vial containing water spiked with OCPs. There is no significant change in the peak areas for each analyte extracted by the FPSE media even after 30 times of recycling as demonstrated in Figure 2h. The results proved that FPSE media are durable and stable with excellent reusability due to the strong bonding between cellulose and sol-gel sorbent coating.

### 2.2. Method Validation

#### 2.2.1. Limit of Detection and Quantification

The proposed method was validated by figures of merit under the optimized experimental conditions (30 min of extraction time, pH 6–7, 9 min desorption time, 5% (*w*/*v*) of NaCl and 400 µL of acetone as desorption solvent agitated at 1200 rpm). The calibration curves were obtained by eight different concentrations of the OCPs’ standard solutions. Good linearity was observed over the wide concentration ranges for the nineteen OPPs with satisfactory determination coefficients (r2). Correlation coefficients (r2) ranging from 0.9929 to 0.9986 were obtained for all the analytes. The LOD and LOQ values were obtained based on a signal-to-noise ratio of 3 and 10, respectively. The instrumental limits of detection (LODs) (S/N = 3) and quantification (LOQs) (S/N = 10) are listed in Table 2. The LODs and LOQs are in the range of 0.007–0.032 ng/mL and 0.023–0.069 ng/mL for all analytes, respectively.

#### 2.2.2. Precision and Accuracy

The precision, in terms of the relative standard deviations was also evaluated by performing five extraction replicates for each of the spiked OCPs at three different concentrations. While the intraday precision was obtained by determining the analytes five times in the same day, and the inter-day precision was obtained by performing the same procedure in five consecutive days. The recoveries and RSD values are shown in Table 2. The intra-day and inter-day RSD values were 2.3–4.6 and 3.3–5.7, respectively, at all concentrations. It is evident from the low RSD values that the developed FPSE/GCMS method is reliable and reproducible for the analysis of OCPs.

#### 2.2.3. Application to Real Samples

**Figure 3:**
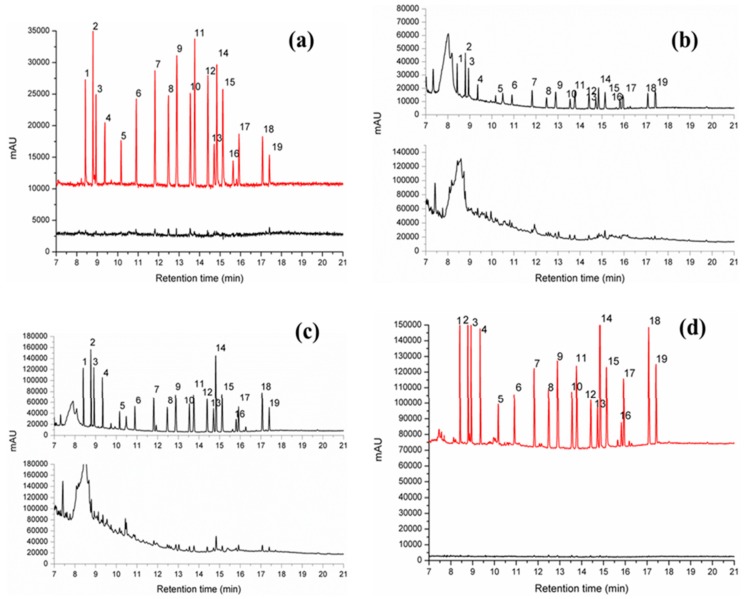
FPSE/GC-MS chromatograms of OCPs in real samples using sol-gel PEG-PPG-PEG coated FPSE media: (**a**) blank and spiked tap water; (**b**) blank and spiked apple juice; (**c**) blank and spiked pomegranate juice; (**d**) blank and spiked litchi juice. [Spiked concentration: 5 ppb].

To examine the applicability of the new FPSE/GC-MS method in real samples, the proposed sol-gel FPSE media coated with sol-gel PEG-PPG-PEG were used to extract and enrich OCPs from water and juice samples. Under the optimized experimental conditions, FPSE media coupled with GC-MS method was validated for the enrichment and determination of OCPs in real samples. The OCP levels in environmental water and drink samples, including tap water, ground water, municipal water, apple juice, pomegranate juice and litchi juice, were analyzed using the FPSE/GC-MS method developed in this study, and the results are summarized in Table 3. Four levels of OCP concentrations (0.1, 1, 10 and 100 ng/mL) were spiked in the actual samples to further test the applicability of the developed method. The spiking recoveries of the target OCPs in the four types of samples are listed in Table 3. The recoveries for the spiked samples ranged from 91.56% to 99.83%. The extracted ion chromatograms of the OCPs acquired from tap water, ground water, municipal water, apple juice, pomegranate juice and litchi juice samples through the developed FPSE/GC-MS method are shown in Figure 3.

### 2.3. Comparison with Other Reported Methods

**Figure 4:**
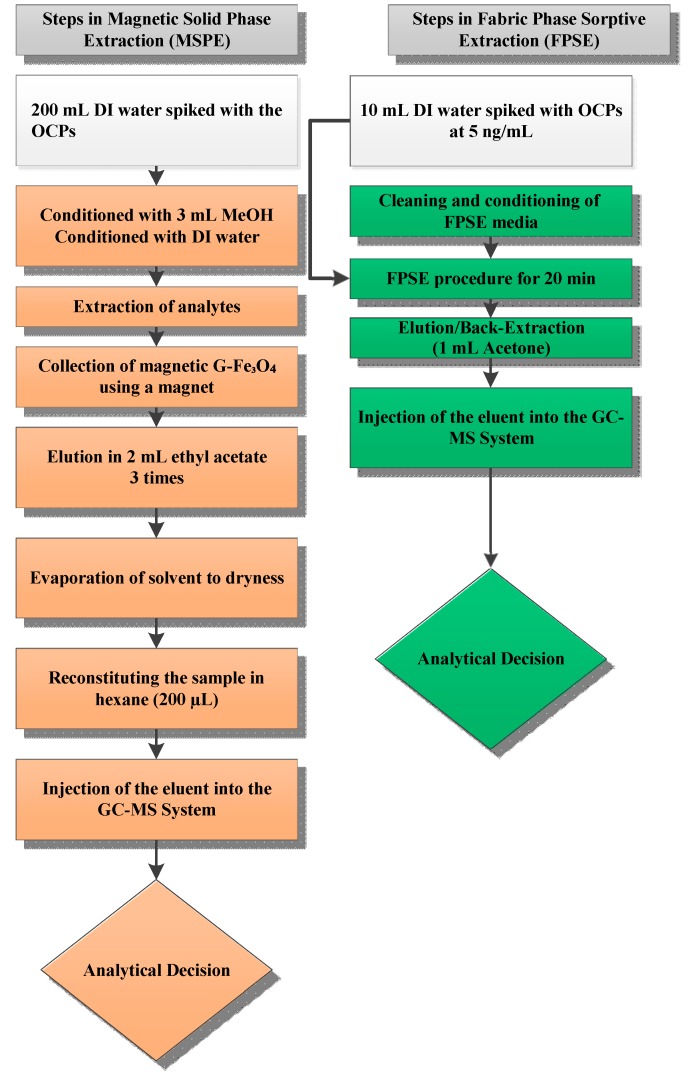
Comparison of sample preparation workflow between magnetic solid phase extraction (**left**) and fabric phase sorptive extraction (**right**).

The capability of the present method was compared with other extraction methods previously reported for the determination of OCPs, with the results being summarized in Table 3. The proposed method demonstrated satisfactory linearity, lower RSD values and comparable recoveries compared with the reported methods. The LOD values of present work were lower than those reported methods (Table 4). FPSE media have a large surface area that greatly increases the contact between analytes and sorbent. This in turn speeds up the extraction process with shorter extraction equilibrium time. Moreover, sol-gel FPSE media was directly inserted into the sample solution for extraction, and hence its usage was very simple. The sponge-like porous architecture of sol-gel sorbent and the capillary action of cellulose fabric synergistically diffuse the organic solvent into the sol-gel sorbent network during back-extraction and allows quantitative recovery of the extracted analytes even when a small volume of organic solvent is used. As such, FPSE also eliminates solvent evaporation and sample reconstitution, often considered to be an integral operation in solid phase extraction. A comparison between magnetic solid phase extraction and fabric phase sorptive extraction workflow is presented in Figure 4. As the schematic demonstrates, FPSE substantially simplifies the sample preparation workflow.

## 3. Material and Methods

### 3.1. Reagents, Solvents and Material

Nineteen certified individual pesticide standards (purity, 96.8–99.55), including α-benzenehexachloride, β-benzenehexachloride, γ-benzenehexachloride, δ-benzenehexachloride, heptachlor, aldrin, heptachlorepoxide, *trans*-chlordane, *cis*-chlordane, p,p dichlorodiphenyldichloroethylene, dieldrin, endrin, β-endosulfan, p,p dichlorodiphenyldichloroethane, endrin aldehyde, endosulfan sulphate, p,p dichlorodiphenyltrichloroethane, endrin ketone and methoxychlor were obtained from Sigma Aldrich (Bangalore, India) and were stored at −4 °C. Individual stock standard solutions (1 mg/L) of OCPs were prepared by dissolving an accurate weight of each pesticide in acetonitrile. Analytical grade methanol, hexane, acetone and acetonitrile were supplied by Merck (Mumbai, India). Water was deionized (Riviera, SCHOTT DURAN, Mainz, Germany) and filtered using 0.45-µm Nylon 6,6 membranes (Rankem, New Delhi, India) filtration assembly (Perfit, India). Working standard solutions were prepared daily by serial dilution of the individual stock solution with acetonitrile. An intermediate stock standard mixture was prepared by mixing the appropriate volumes of individual stock solutions and diluted with highly purified water to a required concentration.

### 3.2. Instrumentation

The pesticide analyses were performed using GC–MS QP 2010 plus (Shimadzu, Kyoto, Japan). Chromatographic separation was conducted with a fused silica capillary column Rtx-5MS, crossbonds 5% diphenyl and 95% dimethylpolysiloxane (30 m × 0.25 mm I.D., film thickness of 0.25 m, J & W Scientific, Folsom, CA, USA). Helium (purity ≥ 99.999%) was used as carrier gas at a constant flow of 1.0 mL/min. The temperature program was set initially at 100 °C for 2.5 min; ramp to 200 °C at a rate of 15 °C/min; 250 °C at 10 °C/min and finally to 300 °C at a rate of 6 °C/min being held for 2 min with total run time of 24 min. Injector temperature was maintained at 280 °C, and the injection volume was 1.0 µL in a splitless mode. Mass spectrometric parameters: electron impact ionization mode with an ionizing energy of 70 eV, injector temperature 250 °C, interface temperatures 230 °C, ion source temperature 200 °C. The mass spectrometer was operated in the selective ion monitoring (SIM) mode and the characteristic ions are given in Table 5. Full-scan data were acquired in the range of *m*/*z* 50–900 to obtain the fragmentation spectra of the analytes. The fragments of the ions monitored in SIM mode were selected based on good selectivity and high sensitivity.

### 3.3. Sample Collection and Preparation

Three types of environmental water samples, including tap water, ground water and municipal water, were collected and analyzed as part of real sample investigation. Tap water was taken from our lab faucet after flowing for 10 min. Ground water samples were collected from the bore well located within Punjabi University Campus, Patiala, Punjab, India in Pyrex borosilicate amber glass containers previously rinsed with triple-distilled water. No previous treatment was conducted for water samples, and all samples were stored at −4 °C in the refrigerator until analysis within 24 h.

The fruit juice (apple, litchi and pomegranate) samples were purchased from a local supermarket (Patiala, India). Fruit juice samples were stored at room temperature before use. Once opened, they were stored in specific food containers at 4 °C and analyzed within 2 days. A 20 mL aliquot of fresh juice was centrifuged at 4000 rpm for 15 min, and then the supernatant was filtered through a 0.45 µm membrane filter into a 50 mL conical flask. Before extraction, 10 mL of filtrate was diluted in a 1:1 ratio with deionized water in a 100 mL volumetric flask. After dilution, 10 mL of fruit juice was used for the extraction by FPSE procedure.

### 3.4. Preparation of Fabric Phase Sorptive Extraction Media

Taking the medium and low polarity of the organochlorine pesticides into consideration, two different sol-gel sorbent coatings, both on 100% cotton cellulose, were prepared and evaluated: sol-gel poly(ethylene glycol)-poly(propylene glycol)-poly(ethylene glycol) (sol-gel PEG-PPG-PEG) and sol-gel poly(caprolactone)-poly(dimethylsiloxane)-poly(caprolactone) (sol-gel PCAP-PDMS-PCAP). Both sol-gel PEG-PPG-PEG and sol-gel PCAP-PDMS-PCAP sorbents are moderately polar. Sol-gel sorbent coating on cellulose substrates involves a series of sequential steps, including (a) substrate selection and surface pre-treatment; (b) design and preparation of the sol solution for the sol-gel sorbent coating preparation of sol solution for coating; (c) sol-gel sorbent coating on the substrate via dip coating process; (d) condition, aging, and cleaning the sol–gel coated FPSE media; and (e) cutting the FPSE media into required size. A detailed procedure for the pre-treatment of cellulose substrate can be found in Kumar et al. [35]. The compositions and molar ratio between sol solution ingredients for sol-gel PEG-PPG-PEG and sol-gel PCAP-PDMS-PCAP, process of sol-gel coating, conditioning and aging as well as the post-coating cleaning protocols are given elsewhere [36]. Briefly, the cellulose fabric was treated first, with 1 M NaOH solution for an hour to eliminate all the residual finishing chemicals and to maximize the number of available hydroxide functional groups that would subsequently binds sol-gel sorbent network to the fabric surface via covalent bonding. The fabric was finally treated with 0.1 M HCl to neutralize any residual NaOH. The sol solution was prepared using the molar ratio sol-gel precursor: organic polymer: acetone: methylene chloride: trifluoroacetic acid: water at 1:0.02:3.26:3.74:1.25:3 for sol-gel PCAP-PDMS-PCAP and 1:0.13:1.94:2.3:0.75:3 for sol-gel PEG-PPG-PEG. The sol-gel sorbent coating on the fabric surface was created via dip coating process by immersing the fabric into the sol solution for 4 h. After sol-gel coating, the fabric was conditioned for 24 h at 50 °C. Subsequently, the coated fabric was cleaned with 50:50 (*v*/*v*) methanol and methylene chloride to remove any un-bonded sol solution ingredients. Finally, after drying, the sol-gel sorbent coated FPSE media were cut into 2.5 cm × 2.0 cm pieces.

### 3.5. Fabric Phase Extraction Procedure

The FPSE media (2.5 cm × 2.0 cm) was rinsed with acetone and then water before use to condition and equilibrate. It was then placed in a glass vial containing 10 mL pure water sample spiked with each of the OCPs at a concentration of 5 ng/mL. The magnetic stirrer was set at 900 rpm for 20 min, with the stirring being provided by a Teflon-coated magnetic stir bar inside the glass vial. After extraction, the FPSE media was removed from the sample solution and dried thoroughly with lint-free tissue. It was immediately placed in a glass vial containing 1 mL desorption solvent (acetone) for 15 min. The extract with target analytes was filtered with syringe filters prior to GC-MS analysis. One microliter of the extract was injected into the GC–MS system. After desorption, the extraction media was washed repeatedly with acetone and water for removing any possible residual analyte or other substances. To do this, the fabric phase media was transferred into 0.5 mL of acetone and then 0.5 mL water for 5 min to remove residual analytes. The carryover effect was randomly tested using 200 µL acetone followed by GC–MS. This examination clearly indicated that the device was reusable, as no analyte peaks were detected. A series of tests proved that the device was reusable up to 30 times without impacting on its extraction efficiency negatively.

## 4. Conclusions

In the present work, we reported the use of a cellulose fabric piece coated with sol-gel extraction sorbent as a sample preconcentration technique with the inherent features of both SPE and SPME methods. Here, the extraction phase, sol-gel PEG-PPG-PEG coated FPSE media with 100% cotton cellulose as the substrate demonstrated the highest affinity for the trace analysis of organochlorine pesticides in aqueous samples. This new sample preparation technique was further coupled to GC-MS for the determination of OCPs in different water and juice samples. Good analytical performance, including accuracy, precision and suitable detection limits with excellent linear dynamic ranges, was obtained under optimized conditions. It is evident from the results that the proposed FPSE/GC-MS methodology proved to be rapid, reliable, sensitive, time efficient, easy to implement using low sample and desorption solvent volume, providing excellent robustness and analytical reproducibility. The technology is expected to have promising potential for the routine analysis of organic pollutants, with the possibility of tuning the most selective sorbent coating based on the target compounds present at trace and ultra-trace level concentrations in various complex matrices.

## Figures and Tables

**Table 1:** Predicted absolute recovery values (%) for selected OCPs on two FPSE media.

FPSE Sorbent	Predicted Absolute Recovery Values (%)						
	β-Endosulfan	Endosulfan Sulfate	γ-BHC	β-BHC	α-BHC	δ-BHC	Endrine Aldehyde
Sol-gel PEG-PPG-PEG	74.49	75.29	76.48	77.65	78.37	84.44	**95.71**
Sol-gel PCAP-PDMS-PCAP	**75.86**	**76.61**	**77.70**	**78.79**	**79.33**	**84.97**	94.88

The highest value obtained for each analyte is presented in bold. As seen in Table 1, the sol-gel PCAP-PDMS-PCAP FPSE sorbent projected higher recovery for almost all of the analytes. Based on this prediction, both FPSE sorbents were subjected to the extraction of OCPs.

**Table 2:** The performance characteristics of the proposed FPSE/GC-MS analytical method.

Organochlorine Pesticides	Linear Range (ng/mL)	Coefficient of Determination, r^2^	LOD (ng/mL)	LOQ (ng/mL)	RSD %	
					Intra-Day	Inter-Day
α-Benzenehexachloride (α-BHC)	0.1–500	0.9977	0.013	0.042	4.2	5.2
β-Benzenehexachloride (β-BHC)	0.1–500	0.9968	0.008	0.026	3.3	4.1
γ-Benzenehexachloride (γ-BHC)	0.1–500	0.9944	0.021	0.069	4.6	5.0
δ-Benzenehexachloride (δ-BHC)	0.1–500	0.9931	0.032	0.105	3.5	4.7
Heptachlor	0.1–500	0.9951	0.014	0.046	4.3	5.4
Aldrin	0.1–500	0.9929	0.026	0.086	2.3	3.8
Heptachlorepoxide	0.1–500	0.9965	0.015	0.049	3.3	4.5
Trans Chlordane	0.1–500	0.9952	0.013	0.042	3.1	3.9
Cis Chlordane	0.1–500	0.9954	0.014	0.046	2.6	3.3
p,p Dichlorodiphenyldichloroeth ylene (p,p’ DDE)	0.1–500	0.9984	0.011	0.0363	2.7	3.7
Dieldrin	0.1–500	0.9963	0.013	0.042	3.5	5.3
Endrin	0.1–500	0.9938	0.012	0.039	3.4	5.6
β-Endosulfan	0.1–500	0.9960	0.016	0.053	2.8	3.7
p,p Dichlorodiphenyldichloroeth ane (p,p’ DDD)	0.1–500	0.9969	0.007	0.023	3.7	4.2
Endrin Aldehyde	0.1–500	0.9977	0.012	0.039	3.1	3.9
Endosulfan sulfate	0.1–500	0.9932	0.015	0.049	4.6	5.5
p,p Dichlorodiphenyltrichloroeth ane (p,p’ DDT)	0.1–500	0.9984	0.021	0.069	2.5	3.9
Endrin ketone	0.1–500	0.9986	0.018	0.059	4.1	5.7
Methoxychlor	0.1–500	0.9971	0.027	0.089	4.4	5.6

**Table 3:** Analytical data obtained from FPSE/GC-MS analysis for the determination of OCPs in water and fruit juice samples.

OCP	Amount Added ng/mL	Tap Water			Ground Water			Municipal Water			Apple Juice			Litchi Juice			Pomegranate Juice		
		Extraction Yield	Intraday RSD (%)	Interday RSD (%)	Extraction Yield	Intraday RSD (%)	Interday RSD (%)	Extraction Yield	Intraday RSD (%)	Interday RSD (%)	Extraction Yield	Intraday RSD (%)	Interday RSD (%)	Extraction Yield	Intraday RSD (%)	Interday RSD (%)	Extraction Yield	Intraday RSD (%)	Interday RSD (%)
α-Benzenehexachloride (α-BHC)	0.1	97.4	4.5	5.1	96.8	4.7	4.9	96.6	3.5	4.8	95.7	4.3	5.4	95.6	3.6	4.4	95.5	4.4	5.6
	1	98.7	4.1	4.9	97.6	4.1	4.8	97.5	3.4	4.6	96.4	4.2	5.1	96.2	3.5	4.7	96.3	4.2	5.2
	10	98.9	3.6	4.3	97.8	4.2	5.2	97.3	3.2	4.2	96.7	3.7	4.8	97.1	3.4	4.3	96.3	3.3	4.9
	100	99.3	3.1	3.9	98.7	3.6	4.1	98.4	2.5	3.1	97.7	3.4	4.6	97.4	2.3	3.5	97.6	3.2	4.5
β-Benzenehexachloride (β-BHC)	0.1	96.4	4.2	5.4	97.4	4.1	4.9	95.6	4.7	5.7	94.4	3.7	5.6	96.9	4.9	5.5	95.5	4.2	5.3
	1	97.7	3.5	5.1	98.7	3.8	4.7	95.5	4.1	3.6	94.9	3.6	4.9	97.2	4.6	5.1	96.4	4.1	5.1
	10	98.4	3.1	4.7	98.6	3.6	4.2	96.3	4.2	3.6	95.3	3	4.5	97.5	4	4.9	96.8	3.3	4.7
	100	99.4	2.9	3.8	99.5	2.5	3.8	98.4	3.6	4.7	96.7	2.8	3.8	98.4	3.9	4.6	97.1	3.1	4.3
γ-Benzenehexachloride (γ-BHC)	0.1	97	3.9	5.1	97.4	3.6	4.7	96.6	4.2	5.3	96.6	4.6	5.5	96.2	4.9	5.7	95.6	4.7	5.7
	1	97.8	3.5	4.9	98.7	3.3	4.1	97.8	3.9	4.2	96.5	3.9	4.3	97.3	4.7	5.1	96.9	4.2	5.2
	10	98.9	3.4	4.3	98.9	3.1	4.2	97.4	3.4	4.8	96.9	3.4	3.9	97.5	3.9	4.6	97.3	3.9	4.9
	100	99.5	2.9	3.9	99.8	2.9	3.4	98.5	2.7	3.6	97.5	3	4.3	98.6	3.5	4.2	97.6	3	4.3
δ-Benzenehexachloride (δ-BHC)	0.1	97.6	3.7	5.3	97.9	3.8	5.1	97.9	4.8	5.8	95.6	3.7	5.1	93.6	4.8	5.6	91.5	4.4	5.1
	1	98	3.4	4.9	98	3.4	4.6	97.6	3.7	5.1	95.9	3.2	4.8	94.2	3.8	4.6	92.3	3.9	4.8
	10	98.7	3.3	4.2	98.6	2.8	3.9	98.5	3.1	4.8	96.4	2.9	3.8	97.1	3.2	4	94.3	3.5	4.1
	100	99.5	3	3.7	99.8	2.4	3.2	98.4	2.9	3.7	96.9	2.1	3.5	98.4	2.7	3.3	96.6	2.7	3.5
Heptachlor	0.1	97.2	3.8	5.1	97.6	4.7	5.7	97.5	4.9	5.9	96.7	3.6	5	97.9	4.6	5.4	97.6	4.4	5.3
	1	97.6	3.4	4.9	97.5	4.1	5	97.8	4.6	5.1	97.4	3.1	4.7	97	3.6	4.4	97.9	3.5	4.3
	10	98.1	3.3	4.3	98.3	4.2	4.9	98.4	4.2	4.9	97.7	2.7	3.9	98.1	3.4	4	98.1	3.3	3.9
	100	99.1	2.9	3.9	99.1	3.6	3.9	98.5	3.6	4.3	98.7	2.2	3.4	98.9	2.9	3.2	98.6	2.8	3.3
Aldrin	0.1	97.6	4.6	5.7	97.4	4.9	5.6	97.6	4.8	5.7	97.7	4.2	4.9	96.9	4.8	5.6	98.1	4.6	5.4
	1	97.6	4.2	5.2	97.2	4.2	4.9	97.9	4.3	5	98.4	3.8	4.5	97.1	4.2	4.9	98.3	3.8	4.1
	10	98.4	3.9	4.3	98.8	3.9	4.2	98.3	4	4.8	98.6	3.3	3.8	97.9	3.9	4.6	99	3.5	3.7
	100	99.3	3.2	3.9	98.5	2.8	3.5	99.4	3.1	4.2	99.4	2.9	3.2	98.1	3.3	3.9	99.6	2.9	3.5
Heptachlorepoxide	0.1	97.5	4.3	5	96.5	3.8	4.6	97.5	4.7	5.6	97	4.1	4.8	96.9	4.7	5.5	97.5	4.5	5.3
	1	98.8	3.6	4.8	97.7	3.3	4	97.6	4.2	5.1	97.2	3.7	4.4	97.1	4.1	5	97.9	3.6	4.2
	10	98.9	3.2	4.2	98.8	3	4.1	98.6	4.1	4.7	98.1	3.4	3.7	97.6	3.6	4.5	98.3	3	3.6
	100	99.1	3	3.8	98.6	2.3	3.5	99.3	2.9	4.1	98.8	2.5	3.1	98.6	3.1	3.9	98.7	2.8	3.4
Trans Chlordane	0.1	97.4	3.8	4.9	97.6	3.9	4.2	97.6	4.6	5.4	97	4.5	5.8	96.6	4.6	5.8	97.5	4.4	5.3
	1	97.9	3.7	4.5	98	3.6	3.9	97.8	4.1	4.9	97.9	3.9	4.4	97.1	4	5.1	98.5	3.7	4.2
	10	98.6	3.6	4.2	98.6	2.7	3.7	98.8	4	4.8	98.2	3.6	3.9	97.4	3.7	4.5	98.7	3.1	3.6
	100	99.2	2.3	3.8	99.8	2.4	2.6	99.4	3.7	4.5	99.6	2.5	3.1	98.3	3.2	3.8	99.1	2.9	3.4
Cis Chlordane	0.1	97.4	3.7	5.1	97.2	4.7	4.7	96.7	4.5	5.3	96.4	4.6	5.5	96.2	4.7	5.7	96.6	4.3	5.2
	1	98.9	3.4	4.9	98.1	4.1	4.1	97.8	4	4.9	96.4	4.3	4.9	97.1	4.1	5	97.4	3.6	4.3
	10	98.7	3.3	4.3	98.6	4.2	4.2	98.5	3.9	4.4	97.2	3.7	4.8	97.8	3.8	4.4	98.6	3.1	3.9
	100	99.3	3	3.9	99.3	3.6	3.6	99.4	2.9	3.5	98.8	2.6	3.2	98.2	3.3	3.1	98.2	2.8	3.5
p,p Dichlorodiphenyldichloroethylene (p,p’ DDE)	0.1	96.5	3.6	5	97.2	4.8	5.2	96.5	4.6	5.6	96.7	4.9	5.6	96.8	4.6	5.5	96.5	4.5	5.1
	1	96.9	3.5	4.8	98.1	4.9	4.3	98	4.2	4.9	96.5	4.4	4.9	97	4	5	97.4	3.5	4.5
	10	97.7	3.2	4.2	98.6	4.7	4.2	98.9	3.8	4.8	97.1	3.9	4.7	97.5	3.9	4.3	98.5	3.1	3.9
	100	98.3	3	3.8	99.3	3.9	3.1	99	2.7	3.5	97.8	2.5	3.3	98	3.4	3.2	98.8	2.7	3.6
Dieldrin	0.1	94.8	4.9	5.8	95.7	4.7	5.6	94.5	4.5	5.5	95.7	4.4	5.5	95.6	4.1	5.3	94	4.3	5.2
	1	95.2	3.7	4.6	96.1	4.2	5.1	95	3.8	4.9	96.6	4.1	5.1	96.4	3.8	4.7	94.9	3.9	4.8
	10	95.6	3.3	4.2	97.2	2.9	3.9	95.8	2.9	3.7	97.1	3.9	4.6	97.6	3.6	4.5	95	3.1	4.2
	100	96.5	2.8	3.9s	98.4	2.6	3.6	96.1	2.5	3.3	97.9	2.8	3.5	98.1	2.3	3.1	96.6	2.6	3.5
Endrin	0.1	96.4	3.9	5.2	97.3	4.7	5.6	95.5	4.5	5.6	95.4	4.7	5.4	96	5.1	5.8	95.6	4.6	5.6
	1	97.1	3.6	4.9	98	4.6	5.2	96.1	4.1	4.8	96.6	4.5	4.9	97.2	4.7	5.2	96.4	3.7	5
	10	97.8	3.3	4.5	98.5	4.7	4.9	97.9	3.9	4.7	97.2	3.9	4.7	97.6	4.2	4.9	97.4	3.2	4.5
	100	98.7	3	3.9	98.7	3.6	4.1	98.5	3.5	4.2	97.8	2.6	3.3	98.1	3.2	3.9	98.7	2.9	3.9
β-Endosulfan	0.1	94.1	4.5	5.6	94.3	4.6	5.8	94.4	4.7	5.4	95.5	4.3	5.4	95.5	4.4	5.3	94.2	4.2	5.1
	1	95.5	3.6	4.7	95.6	4.3	5.2	95.1	4	4.8	96.8	4	5.1	96.3	3.9	4.8	95.9	3.7	4.8
	10	96.2	3.2	4.3	96.7	3.6	4.7	96.8	2.8	3.8	97.3	3.7	4.6	97.4	3.4	4.6	96.9	3.3	4.4
	100	97.8	2.9	3.8	97.8	2.5	3.6	97.4	2.4	3.4	98.8	2.6	3.5	98.6	2.2	3.1	97.2	2.4	3.3
p,p Dichlorodiphenyldichloroethane (p,p’ DDD)	0.1	96.4	3.7	5.3	97.9	4.6	5.5	96.7	4.1	5.5	95.8	4.7	5.8	96.9	4.9	5.6	95.5	4.6	5.5
	1	96.1	3.7	4.9	98.5	4.7	4.9	97.4	4	5.1	96.3	4.2	5.1	97.1	4.2	5.1	95.8	4.1	4.9
	10	97.1	3.4	4.3	98.9	4.5	4.6	97.8	3.8	4.6	97.5	3.7	4.6	97.6	3.7	4.6	96.4	3.8	4.7
	100	98.2	3.2	3.9	99.1	3.7	4.1	98.1	2.5	3.3	97.8	2.7	3.6	98.7	3.2	4.3	97.3	3.1	4.2
Endrin Aldehyde	0.1	95	4.2	4.1	95.2	4.7	5.7	94.1	4.8	5.6	94.1	4.6	5.5	95.2	4.8	5.8	95.8	4.4	5.5
	1	95.8	3.8	4.8	96.5	4.4	5.5	95.4	4.1	5	95.5	4.2	5.1	95.3	3.8	4.7	96.1	4	5.2
	10	96.4	3.1	4.1	97.8	3.5	4.4	95.7	3	4	96.4	3.3	4.5	96.7	3.5	4.4	96.8	3.5	4.7
	100	96.9	2.8	3.5	98.9	2.2	3.1	96.2	2.7	3.5	97.3	2.3	3.2	98.9	2.3	3.2	97.1	2.3	3.2
Endosulfan sulfate	0.1	95.5	4.4	5.6	96.4	4.8	5.6	96.4	4.9	5.8	94.6	5.2	5.9	95.2	5.1	5.9	94.2	4.9	5.8
	1	96.6	3.9	4.9	97.3	4.4	5.2	97	4.6	5.2	95.4	4.5	5.4	96.7	4.8	5.6	95.6	4.4	4.9
	10	97.7	3.5	4.6	98.4	4.1	4.9	97.9	3.2	4.4	96.2	3.9	4.5	97.3	4.1	4.9	96.5	3.9	4.6
	100	98.1	3.2	3.8	99.2	3.5	4.6	98.8	2.7	3.3	97.5	3.2	4.3	98.2	3.4	4.2	97.7	3.3	4.3
p,p Dichlorodiphenyltrichloroethane (p,p’ DDT)	0.1	95.2	3.9	4.6	95.5	4.9	5.8	94.7	4.9	5.8	94.8	4.7	5.6	94.2	5	5.9	94.6	4.8	5.7
	1	96.7	3.4	4.2	96.1	4.6	5.7	95.2	4.2	5.2	95.4	4.1	5	95.5	3.9	5.2	95.8	4.3	5.3
	10	96.1	2.9	3.7	96.8	3.7	4.6	96.5	3.2	4.1	96.3	3.4	4.4	96.6	3.4	4.5	96.1	3.9	4.9
	100	97.5	2.6	3.5	97.7	2.3	3.4	97.5	2.8	3.6	96.9	2.6	3.5	97.6	2.4	3.1	97.7	3	4.2
Endrin ketone	0.1	96.4	3.5	5	96.7	4.8	5.6	95.1	4.2	5.6	94.4	4.6	5.7	95.6	5.3	5.9	95.1	4.8	5.6
	1	97.4	3.1	4.8	97.2	4.6	5.2	96.5	3.8	5.2	95.5	4.1	5.3	95.8	4.4	5.5	95.6	4.3	5.1
	10	98	2.9	3.5	98.2	4.1	4.9	97.8	2.9	4.1	96.9	3.8	4.8	97.1	3.9	4.7	96.6	3.6	4.6
	100	98.9	2.6	3.2	98.8	3.5	4.2	98.3	2.6	3.4	97.2	2.9	3.8	98.1	3.4	4.5	98.1	3	4.1
Methoxychlor	0.1	97	3.4	4.8	95.7	4.6	5.9	95.2	4.1	5.2	94.3	4.8	5.9	95.7	5.1	5.8	95.5	4.9	5.8
	1	97.8	3	4.1	96.2	4.5	5.6	96.6	3.9	5.1	95.4	4.2	5.3	96.1	4.2	5.1	95.5	4.4	5.4
	10	98.2	2.6	3.6	97.2	3.6	4.8	97.2	2.7	3.9	96.8	3.5	4.6	96.8	3.6	4.8	96.1	3.7	4.8
	100	98.8	2.4	3.3	98.8	2.9	4.1	98.7	2.3	3.5	97.3	2.8	3.7	97.2	2.5	3.6	97.6	3.1	4.1

**Table 4:** Performance comparison between FPSE/GC-MS with other reported methods used in preconcentration and determination of organochlorine pesticides analytes.

Sl. No.	Analyte	Matrix	Extraction Method	Chromatographic Technique	Linearity (ng/mL)	LOD (ng/mL)	RSD %	Reference
1	8 OCPs	water	graphene SPE	GC-MS	0.1–10	0.0019–0.0093	<7.4	[14]
2	10 OCPs	Strawberry, strawberry jam, soil	SDME	GC-MS/MS	0.5–50	0.002–0.150	<15	[16]
3	9 OCPs	water	µ-SPE	GC-ECD	0.1–100	0.0076–0.016	<10	[19]
4	14 OCPs	water	HS-SBSE	GC-MS	5–17	0.01–1.59	<14.8	[20]
5	19 OCPs	water and juice samples	FPSE	GC-MS	0.1–500	0.007–0.032	<5	Present work

**Table 5:** The performance characteristics of the proposed FPSE/GC-MS analytical method.

Peak No.	OCP	Molecular Weight	CAS Number	Log Kow	Retention Time (min)	Qualitative Ion
1	α-Benzenehexachloride (α-BHC)	290.83	319-84-6	3.81	8.43	183 *, 219, 109
2	β-Benzenehexachloride (β-BHC)	290.83	31-85-7	3.78	8.79	183 *, 219, 109
3	γ-Benzenehexachloride (γ-BHC)	290.83	58-89-9	3.72	8.94	183 *, 145, 109
4	δ-Benzenehexachloride (δ-BHC)	290.83	319-86-8	4.14	9.35	183 *, 219, 109
5	Heptachlor	373.32	76-44-8	6.10	10.17	100 *, 272, 237
6	Aldrin	364.90	309-00-2	6.50	10.92	66 *, 101, 263
7	Heptachlorepoxide	389.30	1024-57-3	5.40	11.83	53, 81 *, 353
8	Trans Chlordane	409.75	5103-74-2	6.16	12.49	176, 212 *, 375
9	Cis Chlordane	4.9.75	5103-71-9	6.16	12.90	237 *, 272, 373
10	p,p Dichlorodiphenyldichlo roethylene (p,p’ DDE)	318.02	72-55-9	6.51	13.56	176, 246 *, 318
11	Dieldrin	380.91	60-57-1	5.40	13.78	79 *, 263, 108
12	Endrin	380.90	72-80-8	5.20	14.42	81 *, 67, 263
13	β-Endosulfan	406.90	33213-65-9	3.62	14.72	207, 195 *, 159
14	p,p Dichlorodiphenyldichlo roethane (p,p’ DDD)	320.03	72-54-8	6.02	14.85	199, 235 *, 165
15	Endrin Aldehyde	380.89	7421-93-4	4.80	15.15	67 *, 250, 345
16	Endosulfan sulfate	422.90	1031-07-8	3.66	15.82	193, 207 *, 129
17	p,p Dichlorodiphenyltrichlo roethane (p,p’ DDT)	354.48	50-29-3	6.91	15.94	235 *, 165, 199
18	Endrin ketone	380.89	53494-70-5		17.08	67 *, 281, 221
19	Methoxychlor	345.65	72-43-5	5.08	17.42	227 *, 169, 197

* most abundant ion.
